# Jagged-1 is required for the expansion of CD4^+^ CD25^+^ FoxP3^+^ regulatory T cells and tolerogenic dendritic cells by murine mesenchymal stromal cells

**DOI:** 10.1186/s13287-015-0021-5

**Published:** 2015-03-11

**Authors:** Emer F Cahill, Laura M Tobin, Fiona Carty, Bernard P Mahon, Karen English

**Affiliations:** Department of Biology, Institute of Immunology, Maynooth University, National University of Ireland Maynooth, Maynooth, Co. Kildare Ireland

## Abstract

**Introduction:**

Mesenchymal stromal cells (MSC) have well defined immunomodulatory properties including the suppression of lymphocyte proliferation and inhibition of dendritic cell (DC) maturation involving both cell contact and soluble factors. These properties have made MSC attractive candidates for cellular therapy. However, the mechanism underlying these characteristics remains unclear. This study sought to investigate the mechanisms by which MSC induce a regulatory environment.

**Method:**

Allogeneic bone marrow mesenchymal stromal cells were cultured with T cells or dendritic cells in the presence or absence of gamma secretase inhibitor to block Notch receptor signalling. T cells and dendritic cells were examined by flow cytometry for changes in phenotype marker expression. Stable knock down MSC were generated to examine the influence of Jagged 1 signalling by MSC. Both wildtype and knockdown MSC were subsequently used *in vivo* in an animal model of allergic airway inflammation.

**Results:**

The Notch ligand Jagged-1 was demonstrated to be involved in MSC expansion of regulatory T cells (Treg). Additionally, MSC-induced a functional semi-mature DC phenotype, which further required Notch signalling for the expansion of Treg. MSC, but not Jagged-1 knock down MSC, reduced pathology in a mouse model of allergic airway inflammation. Protection mediated by MSC was associated with enhanced Treg in the lung and significantly increased production of interleukin (IL)-10 in splenocytes re-stimulated with allergen. Significantly less Treg and IL-10 was observed in mice treated with Jagged-1 knock down MSC.

**Conclusions:**

The current study suggests that MSC-mediated immune modulation involves the education and expansion of regulatory immune cells in a Jagged-1 dependent manner and provides the first report of the importance of Jagged-1 signalling in MSC protection against inflammation *in vivo*.

**Electronic supplementary material:**

The online version of this article (doi:10.1186/s13287-015-0021-5) contains supplementary material, which is available to authorized users.

## Introduction

Adult mesenchymal stromal (stem) cells (MSC) are multipotent cells that have the ability to differentiate into a number of lineages, including bone, fat and cartilage [1,2]. Primarily, MSC are found in the bone marrow where they provide support for haematopoietic stem cells [3], but they have also been isolated from other tissues [4]. Advances in MSC isolation, culture and differentiation have highlighted MSC as pivotal cells in regenerative medicine. However, it is becoming apparent that MSC therapy is based less on *in situ* differentiation abilities and more on paracrine or trophic factors [5]. MSC can home to sites of injury and induce repair through the release of trophic factors, such as cytokines [6]. One of the major attractions for using MSC as a therapeutic agent lies in the fact that MSC possess an array of immunosuppressive capabilities and can be used in an allogeneic setting. MSC avoid allogeneic rejection through suppressive actions on both the innate and adaptive immune responses [7,8]. However, the precise immunosuppressive signals employed by MSC are not well understood.

The induction and expansion of tolerogenic dendritic cells (tDC) or regulatory T cells (Treg), assist in the maintenance of peripheral tolerance through the active suppression of effector T cell populations, preventing autoimmunity through the activation of self-reactive lymphocytes [9]. This can occur directly through cell-contact mediated suppression of self-reactive effector CD4^+^ T cells by Treg, (infectious tolerance), through the deletion (killing) of effector cells or through the creation of an immunosuppressive environment via the release of regulatory cytokines (bystander suppression) [10,11]. tDC populations typically exhibit an immature or ‘semi-mature’ phenotype, which is defined by low levels of major histocompatibility complex (MHC) and co-stimulatory marker expression, decreased IL12p70 and increased IL-10 production [9,12]. The two main categories of Treg are natural Treg, which develop in the thymus and enter the periphery, and inducible Treg that are induced in the periphery from naïve T cells and aid in the maintenance of tolerance [13]. Both types of Treg can achieve suppression through the production of soluble factors, namely IL-10 and transforming growth factor beta (TGFβ) [14].

Subpopulations of DC in the periphery can induce Treg from naïve CD4^+^ T cells [15,16]. These tDC can present antigen to antigen-specific T cells, but fail to deliver adequate co-stimulation for effector T cell proliferation [9]. A key factor involved in the induction of these DC is IL-10, as the presence of this cytokine can reduce MHC class II expression and IL-12 production [12,17]. tDC expand CD4^+^ CD25^+^ Treg from CD4^+^ CD25^−^ precursors [18], leading to the expansion of antigen-specific Treg which contribute to the prevention of autoimmunity [9,19].

MSC can indirectly induce Treg via the modulation of DC phenotypes [20-23] or directly in the absence of DC [24]. English *et al.* have shown that human MSC expand Treg expressing FoxP3 cells through the release of soluble factors PGE_2_ and TGF-β1, but this study also indicated a role for a cell contact signal [25]. MSC-mediated inhibition of T cell proliferation occurs under proinflammatory conditions and stimulation with IFN-γ induces the production of IDO by MSC [26], now known to play an important role in MSC suppression of T cell proliferation [27,28]. In addition to PGE_2_ and TGF-β1, a requirement for HLA-G5 has also been demonstrated in MSC expansion of Treg, an effect involving IL-10 and cell contact [29]. MSC-induced Treg are functional and play an important role *in vivo*. In an animal model of allergic asthma, MSC were shown to ameliorate pathology and inhibit inflammation through a Treg dependent mechanism [30].

Notch signalling is a well conserved pathway involved in cell fate decisions during development, cell proliferation, differentiation and apoptosis [31,32]. Interactions between Notch receptors and ligands are crucial in the crosstalk between cells of the immune system and their surrounding microenvironment [32]. Notch receptors and ligands are expressed on numerous immune cells including DC and T cells, and the presence of bacterial products, such as lipopolysaccharide (LPS) elevates the expression of Notch ligands on DC [33]. Cheng *et al.* have shown that the Notch ligand Jagged-1, on bone marrow-derived stromal cells, stimulated the accumulation of DC precursors, preventing their transition to terminally differentiated DC. Following exit of the bone marrow, the expression of Delta like ligand-1 on spleen stroma permits full differentiation of DC [34]. In terms of T cell activation and proliferation, Notch signalling plays a key role. Rutz *et al.* demonstrated that Delta-like ligand-1 and Jagged-1 induced partial or complete inhibition of T cell activation, while the expression of Delta like ligand-4 enhanced T cell proliferation [35]. Furthermore, both murine and human naïve CD4^+^ T cells have been differentiated into FoxP3 expressing Treg following TGF-β1 signalling through the Notch pathway [36,37]. Combined, these data provide significant evidence for the involvement of the Notch signalling pathway in the crosstalk between innate and adaptive immune cells and highlights the potential importance of this pathway as a candidate for the contact signal involved in MSC modulation of adaptive immune responses.

This study sought to investigate the importance of the Notch signalling pathway in MSC induction of regulatory DC and CD4^+^ T cells. The Notch signalling inhibitor, gamma secretase (GSI), was used to determine the role of this pathway in MSC-mediated immune regulation using a co-culture system, involving murine MSC, DC or CD4^+^ T cells. Jagged-1, a Notch ligand, was knocked down in murine MSC using shRNA technology. Interestingly, the expression of Jagged-1 was required for the expansion of CD4^+^ CD25^+^ FoxP3^+^ Treg by allogeneic MSC *in vitro.* Allogeneic MSC were capable of disrupting the maturation of DC, inducing a semi-mature tolerogenic DC phenotype. Additionally, these tolerogenic DC induced by MSC were capable of inducing Treg from a population of CD4^+^ CD25^−^ FoxP3^−^ cells. These data confirmed that allogeneic MSC are capable of regulatory cell expansion *in vitro* and that this process requires Jagged-1 signalling. Utilising a mouse model of allergic airway inflammation, this study demonstrated the important role played by Notch signalling and specifically MSC expression of Jagged-1 in MSC-mediated protection against inflammation *in vivo*. MSC, but not Jagged-1 knock down MSC, reduced allergen driven airway pathology. The abrogation of protection observed with Jagged-1 knock down MSC was associated with decreased Treg in the lungs and reduced IL-10 production in the spleen. Importantly, this study demonstrates a role for direct cell to cell contact via the Notch signalling pathway in the development of a regulatory environment by MSC both *in vitro* and *in vivo*.

## Materials and methods

### Animals

Six- to eight-week old female BALB/c, C57BL/6 (Harlan, BiMA, Chester, Oxon, UK), BALB/c-Tg (DO11.10)10Loh/J or eGFP-FoxP3 (The Jackson Laboratories, Bar Harbour, Maine, USA) were used for cell based experiments under the guidelines of the Irish Department of Health and the approval of the research ethics committee of the National University of Ireland Maynooth. Allergen sensitization was carried out as previously described [38] using eight- to twelve-week old female BALB/c mice. Mice were maintained according to the regulations of the Irish Department of Health, and the institutional research ethics committee. Mice were sensitized by intra-peritoneal injection of 33 μg/mouse ovalbumin (OVA) emulsified in aluminium hydroxide (AlumImject™) (Pierce, Rockford, IL, USA) on days 0, 7 and 14. Mice were challenged intra-nasally with OVA (50 μg/mouse) or sterile PBS on days 14, 25, 26 and 27. BALB/c derived MSC or Jagged-1 knock down MSC (generated as described below) were administered intravenously in sterile PBS (0.5 × 10^6^/mouse) on days 7 and 14.

### Histopathology

On day 28, lungs were harvested, fixed and paraffin embedded followed by sectioning and staining with haematoxylin/eosin (H & E). For analysis of airway pathology, at least five fields of view were examined in five sections from five different mice per group. Pathology was scored using a semi-quantitative scoring system as mild, moderate or severe.

### Lung digestion and preparation of single cell suspension for analysis of Foxp3

On day 19, lungs were harvested and homogenised using a tissue homogeniser and centrifuged at 1,500 RPM for five minutes. The tissue pellet was then digested with 100 U/ml collagenase (Sigma Aldrich, Arklow, Ireland) and 20 U/ml DNase (Roche, Dublin, Ireland) in 1 ml PBS at 37°C for one hour. The tissue was then passed through a 70 μm pore cell strainer and resuspended in 25 ml RPMI and overlayed on 15 ml lymphocyte separation media (PAA, London, UK). A density gradient centrifugation step was then performed at 2,400 RPM for 25 minutes without the brake at room temperature (RT). The buffy layer consisting of lymphocytes was removed for counting and staining for Foxp3 as described below.

### Measurement of IL-10 in splenocytes re-stimulated with ovalbumin

Spleens were harvested on day 19 and a single cell suspension was prepared by dissociating spleens and passing through a 70 μm pore cell strainer before centrifugation and subsequent red blood cell lysis. Splenocytes were counted and cultured at 1 × 10^5^/well in triplicate in complete RPMI in a 96-well round bottomed plate. Spleen cells were re-stimulated with OVA (200 μg/ml) for 48 hours. Supernatants were harvested and analysed in triplicate for IL-10 by ELISA (Peprotech, London, UK) according to the manufacturer’s instructions.

### Isolation and characterisation of bone marrow derived mesenchymal stem cells

Murine MSC were isolated from the bone marrow of BALB/c or C57BL/6 mice as previously described [28]. Briefly, following the isolation of bone marrow, MSC were cultured in complete RPMI, supplemented with 10% (v/v) heat inactivated fetal bovine serum (FBS) (Invitrogen-Gibco, Paisley, Scotland), 10% (v/v) equine serum (Hyclone Laboratories, Logan, UT, USA), 1% (v/v) penicillin/streptomycin (Invitrogen-Gibco,), and 1% (v/v) L-glutamine (Invitrogen-Gibco) for 28 days, replacing with fresh media every three to four days. At passage 2, MSC were expanded in complete alpha MEM supplemented with 10% (v/v) heat inactivated FBS, 10% (v/v) equine serum, 1% (v/v) penicillin/streptomycin and 1% (v/v) L-glutamine. MSC were analysed for the expression of a variety of fluorescein isothiocyanate (FITC) or phycoerythrin (PE) conjugated surface markers including Sca-1, MHC class I, MHC class II, CD11b, CD11c, CD34, CD44, CD45, CD80, CD86, CD90, CD105, CD106 and CD117 (eBiosciences, San Diego, CA, USA) by flow cytometry (BD FACS Calibur and CellQuest software) at every passage (Additional file 1: Figure S1A). The ability of MSC to suppress allogeneic and mitogen-driven proliferation was also assessed at each passage (Additional file 1: Figure S1B). The differentiation capacity of MSC following the addition of differentiation media [25] into osteocytes, adipocytes and chondrocytes were analysed at every passage (Additional file 1: Figure S1C,D). MSC used in these studies expressed MHC class I, Sca-1, CD44 and CD106 and differentiated into all three lineages mentioned above. MSC were used between passage three to ten.

### shRNA

Five Jagged-1 short hairpin RNA (shRNA) plasmids were expressed in the lentiviral vector pGIPZ (Open Biosystems-Thermo, Dublin, Ireland). Jagged-1 plasmids were transfected into HEK293t cells along with the packaging plasmid Trans-Lentiviral packaging mix (Open Biosystems, (Fisher Scientific), Dublin, Ireland) using Gene Juice (Merck, Millipore, Darmstadt, Germany). HEK293t cells were cultured in high glucose (D)MEM (Sigma Aldrich, Arklow, Ireland) supplemented with 10% (v/v) heat inactivated FBS, 1% (v/v) penicillin/streptomycin (Invitrogen, (Life Technologies), Paisley, UK) and 1% (v/v) L-glutamine (Invitrogen); during transfection they were cultured in serum free (D)MEM without antibiotics. After 24 hours transfection, they were switched to 30% FBS. The supernantant containing the Jagged-1 shRNA lentivirus was collected 48 hours after transfection. BALB/c MSC were transduced with the viral medium for 72 hours followed by positive selection using 4 μg/ml puromycin. shRNA knock down cells were characterised as above and expression of Jagged-1 was examined by real-time PCR to ensure stable knock down following sub-cloning (Additional files 2 and 3: Figures S2 and S3).

### Co-culture experiments: MSC, DC and T cells

Immature DC were isolated from the bone marrow of BALB/c or C57BL/6 mice and cultured in complete RPMI (containing 10% (v/v) heat inactivated FBS, 1% (v/v) penicillin/streptomycin and 1% (v/v) L-glutamine) supplemented with 20 ng/ml of recombinant murine Granulocyte monocyte-colony stimulating factor (GM-CSF), cell culture medium was replenished on day 3 and 6. Immature DC were harvested on day 8 and matured (0.5 × 10^6^/ml) in the presence of LPS (100 ng/ml) (Sigma-Aldrich) for 48 hours in the presence or absence of bone marrow derived MSC (1.5 × 10^5^/ml) in a six-well plate (Final Ratio 3:1 DC:MSC). The expression of maturation markers, MHC II and CD86 was analysed by flow cytometry on DC using BD FACS Calibur and CellQuest software or BD Accuri C6 and corresponding software (Additional file 4: Figure S4A,B).

#### CD4^+^ T cell proliferation

Immature BALB/c DC (0.5 × 10^6^/ml) were pulsed with OVA (20 μg/ml) (Sigma-Aldrich) in the presence or absence of allogeneic MSC (1.5 × 10^5^/ml) or GSI (1 μM) for 24 hours in a six-well plate (Final Ratio 3:1 DC:MSC). DC were harvested from adherent MSC via gentle aspiration and further cultured with naïve OVA-specific I-A^d^ restricted CD4^+^ T cells (4 × 10^5^/ml) (Final Ratio 4:1 T:DC) isolated from DO11.10 murine spleens using MagCellect (R & D Systems, Abingdon, UK) for 72 hours. Proliferation was analysed using thymidine (5 μCi/ml) incorporation quantified as mean counts per minute (± SE) by liquid scintillation (1,450 Microbeta Liquid Scintillation counter, Wallac-Perkin Elmer, Dublin, Ireland).

#### FoxP3 detection

C57BL/6 T cells were isolated by positive selection for CD4 using magnetic-activated cell sorting (MACS) beads (Miltenyi Biotech, Surrey, UK) or by cell sorting for CD4^+^CD25^−^FoxP3^−^ cells. Subsequently, sorted T cells (1 × 10^6^/ml) were cultured in the presence or absence of MSC or Jagged-1 knock down MSC (3.3 × 10^5^/ml) or GSI (1 μM) for 72 hours in a six-well plate (Final Ratio 1:3 MSC:T cells). CD4^+^ regulatory T cells were detected by expression of GFP-FoxP3 and CD25 (eBioscience) by flow cytometry. Preparation for Foxp3 detection in separated cells and *ex vivo* tissues (lungs) was performed using a Foxp3 Staining Buffer Set (eBioscience) according to the manufacturer’s instructions.

### Statistics

Statistical analysis was performed using GraphPad Prism™ software (GraphPad, San Diego, CA, USA). The student’s paired t-test was used when statistical analysis was required between two experimental groups. One way analysis of variance (ANOVA) was used to test for statistical significance of differences when multiple experimental groups were compared. Data are presented as the ± standard error of the mean (SEM). *P*-values of *P* <0.05 (*), *P* <0.01 (**) or *P* <0.001 (***) were considered statistically significant.

## Results

### Notch signalling is required for MSC expansion of regulatory T cells

**Figure 1:**
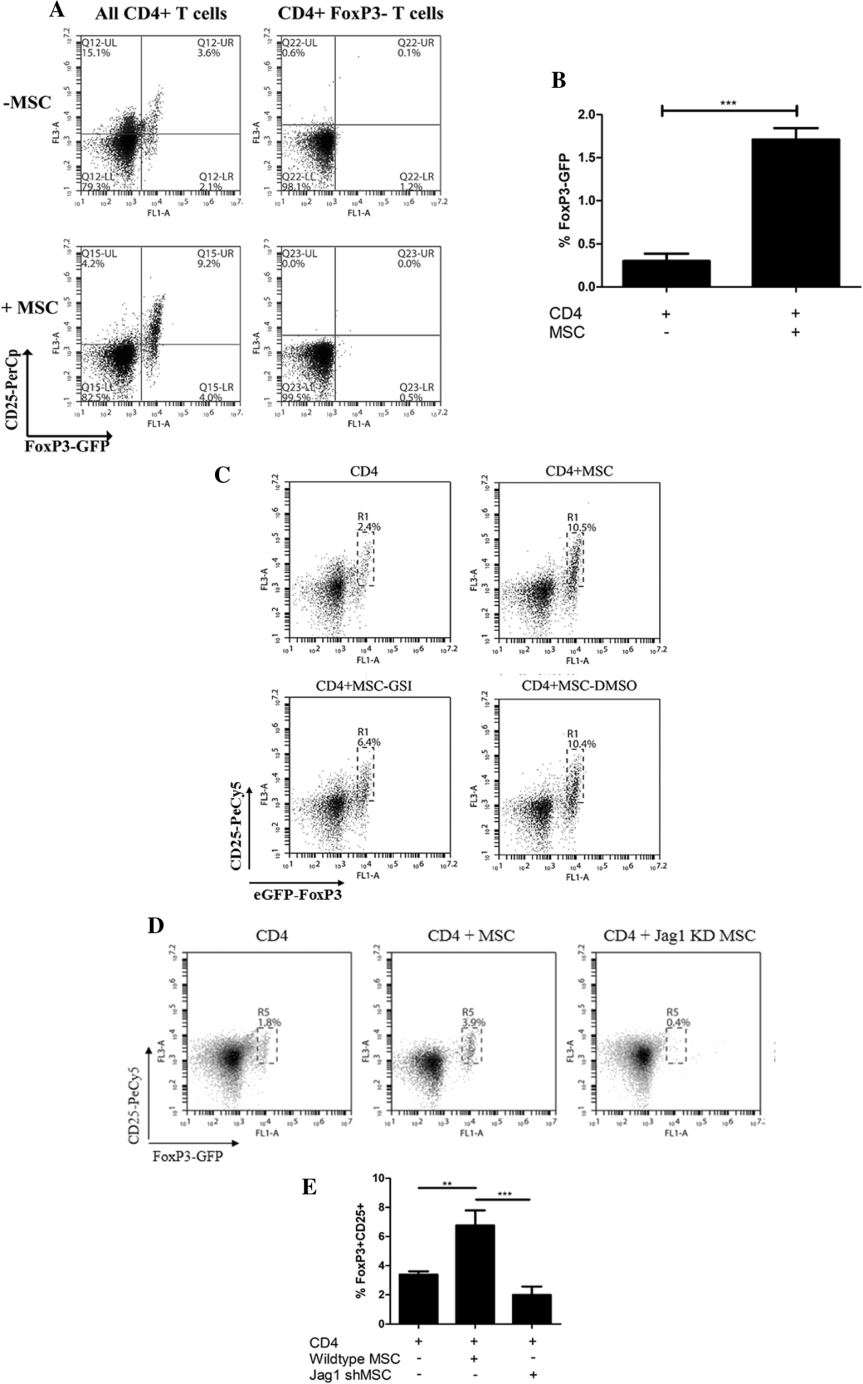
**MSC promote the expansion of regulatory T cells in a Notch dependent manner, specifically through Jagged 1 signalling.** T cells from eGFP-FoxP3 transgenic mice were isolated by magnetic bead isolation (CD4) or cell sorting (CD4^+^ CD25^−^ FoxP3^−^), and subsequently cultured with or without MSC for 72 hours and examined for CD25 and FoxP3-GFP expression by flow cytometry. Culture of CD4^+^ CD25^−^ FoxP3^−^ cells cultured with MSC did not result in an increase in regulatory T cells whereas co-culture of CD4^+^ T cells with MSC resulted in expansion of the existing regulatory population **(A,B)**. MSC significantly increased the expansion of Treg cells, compared to CD4 cells cultured alone (***, *P* <0.001), bar chart displaying n = 7 **(B)**. To examine the role of Notch, CD4^+^ T cells were cultured with MSC in the presence or absence of GSI (1 μM) or vehicle control DMSO and examined for CD25 and GFP expression by flow cytometry **(C)**. CD4^+^ T cells were cultured with wild type MSC or Jagged-1 knockdown MSC for 72 hours and then examined for CD25 and FoxP3-GFP expression by flow cytometry **(D)**. Counting beads were used to determine the number of cells expressing both CD25 and FoxP3 **(E)**. Data are representative of three studies. DMSO, dimethyl sulfoxide; GSI, gamma secretase inhibitor; MSC, mesenchymal stromal cells; Treg, regulatory T cells.

Previous data published by our group demonstrated that MSC are capable of expanding Treg both *in vitro*, using a co-culture system to establish the importance of soluble factors released by MSC, and *in vivo*, using an OVA induced model of allergic asthma [25,30]. Neither study however, determined whether MSC were expanding the native Treg population or inducing Treg from naïve T cells. To investigate this, CD4^+^ T cells were isolated from an eGFP-FoxP3 knock-in mouse using separation beads and sorted for two populations of cells, CD4^+^ CD25^+^ FoxP3-GPF^+^ or CD4^+^ CD25^−^ FoxP3-GFP^−^. In these mice, the FoxP3 transcription factor is co-expressed with eGFP, allowing for specific detection of FoxP3 solely from the spleen cells isolated from these mice. This is an advantage when working with MSC as previous studies have demonstrated expression of FoxP3 in MSC; however, this was not replicated in our hands (data not shown). When the CD25^−^ FoxP3^−^ T cells were co-cultured with allogeneic MSC, GFP-FoxP3 was not detected, suggesting that MSC did not induce the expression of FoxP3 on CD4^+^ CD25^−^ T cells *in vitro* (Figure 1A). However, following co-culture of MSC with CD4^+^ CD25^+^ FoxP3^+^ T cells for 72 hours, the expression of both CD25 and FoxP3 was increased, indicating that MSC were involved in the expansion of existing FoxP3^+^ Treg (Figure 1A,B).

To investigate the role of Notch signalling in MSC expansion of CD4^+^CD25^+^FoxP3^+^ Treg, CD4^+^ T cells were co-cultured with MSC in the presence or absence of a Notch signalling inhibitor (GSI). MSC cultured with purified CD4^+^ T cells supported the expansion of CD4^+^CD25^+^FoxP3^+^ T cells. However, in the presence of GSI, the percentage of cells expressing both CD25 and FoxP3 was reduced (Figure 1C). The vehicle control, DMSO, had no effect on the expansion of Treg. These data suggested that MSC expansion of CD4^+^CD25^+^FoxP3^+^ Treg required functional signalling through the Notch pathway.

MSC expressed high levels of Notch-2, Jagged-1 and Delta like ligand-1 and lower levels of Notch-1 and Jagged-2 (Additional file 2: Figure S2). Previously published data on the Notch ligand Jagged-1 suggested a role for this ligand in the induction of Treg. Overexpression studies showed that activation of the Notch pathway on CD4^+^ T cells by Jagged-1 resulted in increased expression of CD25 and FoxP3 [39]. Given the important role for Jagged-1 in Treg induction and the high expression by MSC, Jagged-1 was chosen for targeted inhibition studies. Jagged-1 expression on MSC was stably knocked down using lentiviral shRNA (Additional file 2: Figure S2). Jagged-1 knock down MSC were used in co-culture with CD4^+^ T cells to determine the role for Jagged-1 in MSC driven expansion of Treg. CD4^+^ T cells were isolated from eGFP-FoxP3 mice and co-cultured with allogeneic wild-type MSC, or Jagged-1 knock down MSC (Figure 1D,E). In the absence of MSC, the CD4^+^ T cell population contained low levels of FoxP3^+^ cells after three days. Wild-type MSC supported the expansion of CD4^+^CD25^+^ FoxP3^+^ T cells whereas Jagged-1 knock down MSC were unable to expand CD4^+^FoxP3^+^CD25^+^ Treg (Figure 1D,E). These data demonstrated that the ability of MSC to expand Treg required Jagged-1 signalling. A caveat to interpreting these results lies in the non-silencing control. This plasmid is typically used where an empty vector is packaged into viral units and the MSC are transduced to ensure there are no unintentional side effects to the viral transduction. In the case of MSC, the non-silencing control delivered by means of a lentivirus may stimulate the TLR3 receptor which is responsible for detecting viral double stranded DNA. TLR3 stimulation on MSC results in the down regulation of Jagged-1 at both the mRNA and protein level [40]; for this reason the non-silencing control could not be used as a valid control for lentiviral transduction in this case. In an effort to overcome this, reverse transcriptase PCR was also performed on lentiviral treated MSC to ensure that knock down of Jagged-1 did not interfere with expression of the other Notch ligands. Additional file 2: Figure S2D demonstrates that Delta like ligand-1 (Dll1) expression is not affected in both Jagged-1 KD MSC and non-silencing control MSC. These results confirm that treatment of MSC with Jagged-1 shRNA results in significantly reduced expression of Jagged-1 at both an mRNA level and a protein level. Although non-silencing control cells also showed reduced expression of Jagged-1, there was no change in the expression of another Notch ligand, Dll1. Taken together it can be concluded from these results that any effects seen from shRNA treated MSC were a consequence of eliminating Jagged-1 signalling.

### MSC induction of functional tolerogenic dendritic cells required Notch signaling

**Figure 2:**
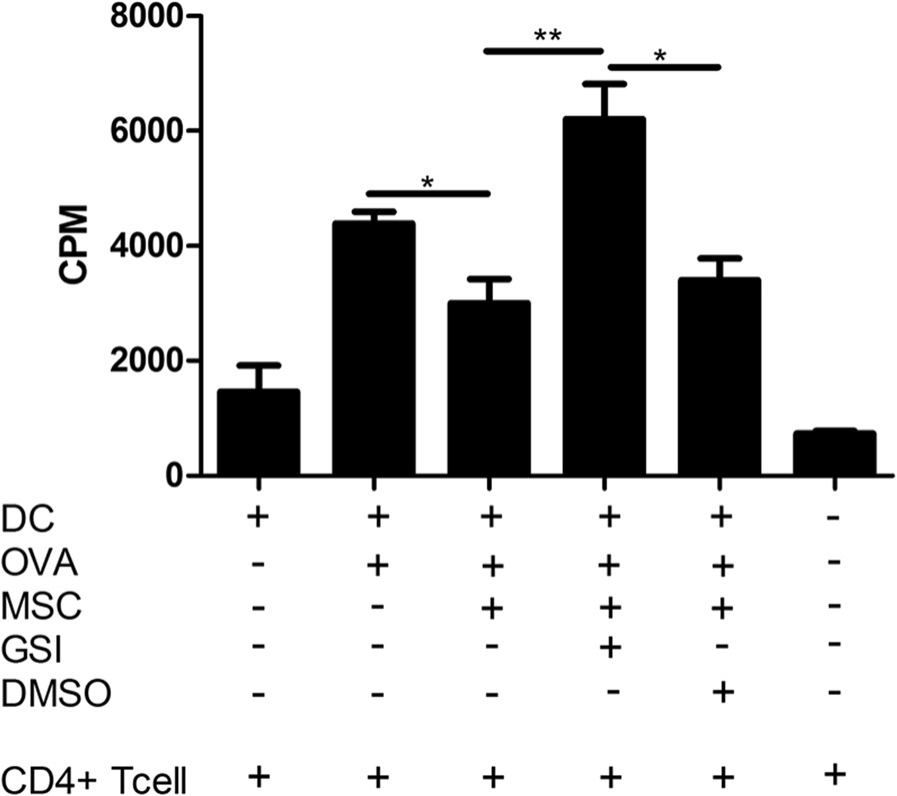
**Notch is required for the induction of functionally tolerogenic DC by MSC.** A two-stage proliferation assay was carried out. Stage one: BALB/c DC (0.5 × 10^6^/ml) were pulsed with OVA (20 μg/ml) in the presence or absence of allogeneic MSC (1.5 × 10^5^/ml), GSI (1 μM) or DMSO (vehicle control) for 24 hours. Stage two: DC were harvested and placed into a second proliferation assay with DO11.10 CD4^+^ T cells (1:4, 1 × 10^5^ DC: 4 × 10^5^/ml T cells for 72 hours. Proliferation was analysed using thymidine (5 μCi/ml) incorporation quantified as mean counts per minute (± SEM) by liquid scintillation. Data represent three studies (*, *P* < 0.05; **, *P* < 0.01). DC, dendritic cells; DMSO, dimethyl sulfoxide; GSI, gamma secretase inhibitor; MSC, mesenchymal stem cells; OVA, ovalbumin; SEM, standard error of the mean.

Murine MSC suppress the maturation, migration and antigen presentation of DC *in vitro* ([21] and Additional file 4: Figure S4A,B) leading to the generation of a tDC population. Although wild-type MSC prevented DC antigen presentation, knocking down Jagged-1 in MSC partially reversed this effect (Additional file 4: Figure S4C). The importance of Notch signalling in allogeneic MSC induction of tDC was investigated using a co-culture system. BALB/c DC were pulsed with OVA in the presence or absence of C57BL/6 MSC. DC were then harvested by gentle aspiration from adherent MSC and cultured with OVA specific DO11.10 CD4^+^ T cells. DC pulsed with OVA that had not encountered MSC, were capable of supporting DO11.10 CD4^+^ T cell proliferation (Figure 2). In contrast, DC pulsed with OVA in the presence of MSC were significantly less proficient in supporting CD4^+^ T cell proliferation (Figure 2) indicating that MSC encounter impairs DC capacity to promote T cell proliferation.

The role of Notch signalling in this interaction was also examined using the two stage assay. Following co-culture with MSC and GSI, OVA pulsed DC were re-purified by gentle aspiration and re-cultured with DO11.10 CD4^+^ T cells. Interestingly, by blocking Notch signalling through the addition of GSI (but not the vehicle control, DMSO), MSC were no longer able to induce functional tDC, as indicated by a significantly increased CD4^+^ T cell proliferation (**, *P* <0.01) (Figure 2). In addition, the proliferation of OVA-specific T cells increases over the baseline when DC were primed in the presence of MSC and GSI. This suggests that removing the suppressive capability of MSC during DC priming, uncovers an allogeneic stimulus. These data confirmed that Notch signalling was required for the induction of a functional tDC population by MSC *in vitro*.

### MSC educated tolerogenic dendritic cells induced regulatory T cells *in vitro*

**Figure 3:**
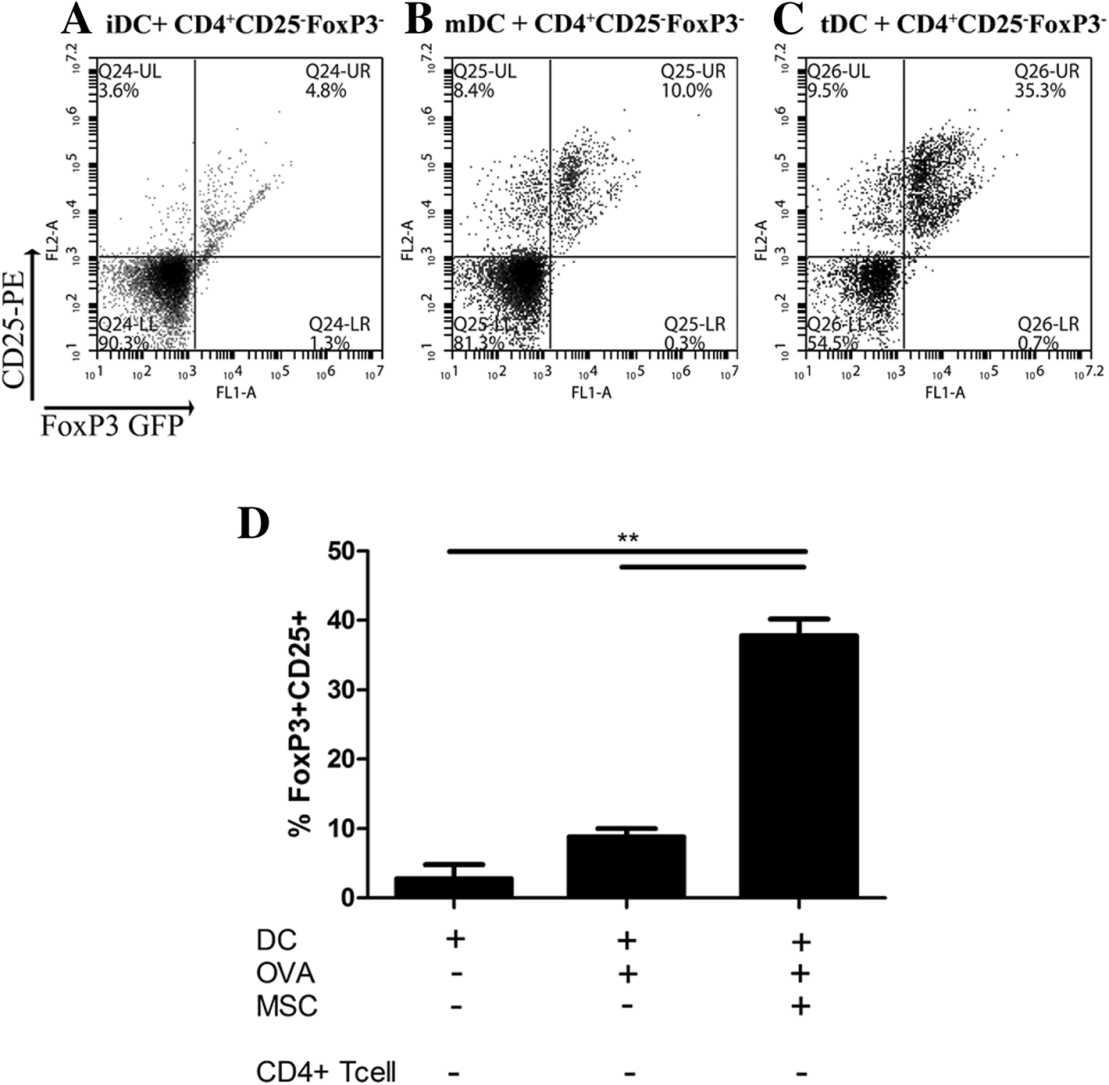
**MSC educated tolerogenic DC induce regulatory T cells.** C57BL/6 DC were cultured in the absence (immature DC) or presence (mature DC) of LPS without or with MSC (tolerogenic DC), and harvested from adherent MSC via gentle aspiration after 48 hours. These DC cells were washed and subsequently cultured with sorted CD4^+^CD25^−^FoxP3^−^ T cells at a ratio of 1:4 DC to T cell, for 72 hours. T cells were gated on CD3 to discriminate them from the DC. Expression of CD25 and FoxP3-GFP was examined by flow cytometry following co-culture with immature DC **(A)**, mature DC **(B)** or tolerogenic DC **(C)**. MHCII^low^ CD86^low^ tolerogenic DC significantly increased the induction of Tregs compared to immature DC or mature DC (**, *P* <0.01) **(D)**. Data in figures A, B and C are representative flow cytometry plots while figure D shows statistics from three studies. DC, dendritic cells; LPS, lipopolysaccharide; MHC, major histocompatibility complex; MSC, mesenchymal stromal cells.

Induction of Treg could account for the inhibition observed in the proliferation assay. Therefore, the influence of MSC on DC driven induction of Treg was studied. C57BL/6 DC were co-cultured with allogeneic MSC in the presence or absence of LPS. TLR stimulation of MSC has been shown to alter both the expression of immune modulatory factors and the immune suppressive function of MSC [40-42]. However, these findings are conflicting, with some studies showing that TLR activation reduces, or others that it enhances, MSC immune suppressive effects. In addition, these studies examining LPS stimulation of MSC focus on MSC effects on T cell proliferation. After 48 hours, DC were recovered by gentle aspiration from adherent MSC and subsequently cultured with sorted FoxP3-eGFP^−^ CD25^−^ T cells. DC matured with LPS (mDC) induced two fold more FoxP3^+^ Tregs than immature DC (iDC). In line with this, studies have shown that LPS-matured DC secrete anti-inflammatory cytokines (IL-10) and promote the induction of Treg [43-45]. Interestingly, DC that had previously been co-cultured with MSC and expressed MHC ClassII^low^ and CD86^low^ cells markers (tDC) induced greater numbers of FoxP3^+^ Treg (approximately seven fold more than iDC). These data provided strong evidence to suggest that MSC educated DC promote a regulatory environment (Figure 3).

### MSC therapy, but not Jagged-1 knock-down MSC therapy, reduced allergen-driven airway pathology and increased Treg in the lungs in an animal model

**Figure 4:**
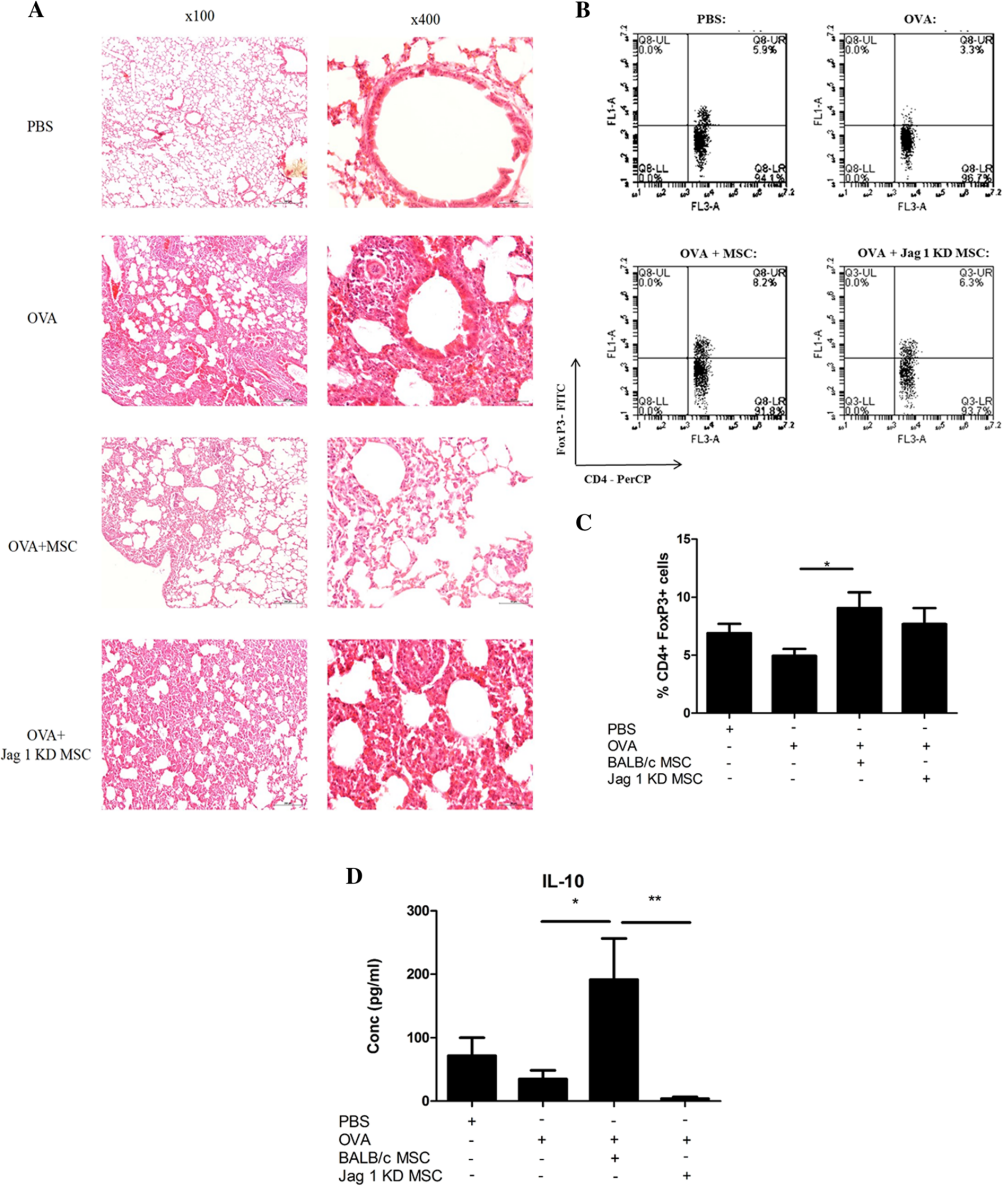
**Jagged 1 knockdown abrogates MSC protection in an OVA driven mouse model of allergic airway inflammation.** Representative images of H & E staining (magnification x100 (left column) and x400 (right column)) from OVA sensitized mice at day 28. MSC, Jag 1 KD MSC or PBS was administered intravenously on day 7 and day 14 **(A)**. A single cell suspension was isolated from the lung at day 19 and analysed by flow cytometry. CD4^+^ cells were gated and examined for FoxP3 expression **(B)**, summarised as % positive CD4^+^FoxP3^+^ cells **(C)**. Spleen cells isolated at day 19 were cultured and re-stimulated with 200 μg/ml OVA for 48 hours. Supernatant was collected and IL-10 expression determined by ELISA **(D)** n = 5. (*, *P* <0.05; **, *P* <0.01). KD, knockdown; MSC, mesenchymal stromal cells; OVA, ovalbumin.

Previously, we demonstrated the capacity for MSC to prevent allergic airway inflammation and discovered a key role for Treg in this setting [30]. A standard murine model of allergic pathology was utilised to investigate the importance of Jagged-1 signalling in MSC protection against allergen-driven airway pathology. In line with our previous findings, MSC therapy reduced pathology and decreased peribronchial inflammation (Figure 4A) in comparison to OVA-sensitized mice without MSC treatment (Figure 4A). In contrast, treatment with Jagged-1 knock down MSC failed to prevent peribronchial inflammation and bronchial epithelial hypertrophy (Figure 4A). Given that MSC protection in this model of allergic airway inflammation has been shown to require Treg, the presence of Treg in the lungs of these mice was examined *ex vivo* using flow cytometry. Notably, a significant increase in the proportion of Foxp3^+^ CD4^+^ T cells was observed in the lungs of MSC treated compared to untreated OVA-sensitized mice (Figure 4B) consistent with previous observations [30,46]. However, there was no significant increase in Foxp3^+^ CD4^+^ T cells in lungs from Jagged-1 KD MSC treated OVA-sensitized mice (Figure 4B,C).

MSC may promote immune suppression through enhancing secretion of anti-inflammatory cytokines such as IL-10. To test this hypothesis, spleen cells from OVA sensitized and MSC treated or untreated mice were harvested and re-stimulated with OVA *ex vivo*. IL-10 production by spleen cells post OVA re-stimulation was measured by ELISA. Enhanced IL-10 levels were observed in spleen cell supernatants from MSC treated mice and this was significantly reduced in the Jagged-1 knock down MSC treated group (Figure 4D). Although previous studies have demonstrated the capacity for MSC to enhance macrophage derived IL-10 *in vivo* [47], the increase in the proportion of Treg and production of IL-10 by spleen cells suggests that IL-10 was produced by Treg in this case. These data suggest that Jagged-1 is involved in MSC promotion of Treg and IL-10 production at the site of allergen challenge and in the secondary lymphoid organs, respectively. Moreover, this study combined with our previous data [30] supports the idea that MSC require Jagged-1 to promote the expansion of functional Treg *in vivo*, which have previously been demonstrated to be required in MSC protection in allergic airway-induced inflammation [30].

## Discussion

The present study sought to demonstrate that allogeneic murine bone marrow derived MSC modulate a regulatory environment through the Notch signalling pathway. Global inhibition of Notch signalling using GSI and targeted inhibition of Jagged-1 signalling through shRNA knock down resulted in abrogation of MSC regulatory T cell expansion. While DC that encountered MSC displayed a ‘semi-mature’ phenotype capable of inhibiting antigen specific T cell proliferation*,* these MSC-induced DC were also capable of Treg expansion. Although the expansion of a Treg population is a known consequence of tDC and T cell co-culture, to our knowledge this is the first time tDC generated in the presence of MSC have been shown to induce Treg from a CD4^+^Foxp3^−^ population.

Interactions between Notch receptors and ligands play a crucial role in the cross talk between cells of the immune system and their surrounding microenvironment [32]. The expression of Notch receptors and ligands are important in stem cell maintenance, cell proliferation, differentiation, apoptosis and induction of peripheral tolerance [31,32,48]. English *et al*. previously demonstrated a role for both soluble factors, PGE-2 and TGF-β1, and an unknown cell contact signal required for MSC expansion of Treg *in vitro* [25]. Recently Kavanagh *et al*. explored the significance of MSC expansion of Treg in an *in vivo* model of allergic airway inflammation. Using an OVA driven model of allergic airway inflammation, MSC were shown to reduce lung pathology and cell infiltration through expansion of a Treg cell population, an effect that was abrogated when Treg populations were depleted [30]. MSC inability to induce Treg (in this system) from a FoxP3^−^ population infers a role in the expansion of Treg rather than the education of naïve T cells.

It is well documented that MSC are capable of expanding a Treg population, both *in vitro* and *in vivo*. While many studies describe the importance of soluble mediators, less is known about the cell contact signal required [25,49,50]. Numerous studies have suggested a role for Notch signalling in the induction of Treg in general [39,51-53]. Yvon *et al*. demonstrated that antigen presenting B cells overexpressing Jagged-1 were capable of reducing IFN-γ, IL-5 and IL-2 secretion from naïve T cells, while upregulating the production of TGF-β, a soluble factor associated with a Treg phenotype [39]. In support of these findings, Asano *et al*. showed that both the Notch and TGFβ signalling pathways interact together, with the blockade of the Notch signalling pathway interfering with the suppressor effect mediated by the production of TGF-β by Treg [51]. In addition, both Notch-1 and TGFβ have been shown to regulate the expression of FoxP3 and the induction of a Treg population [53]. Taken together, these findings highlighted an important role for Notch signalling in Treg induction/expansion further explored herein.

Notch has been previously implicated as a contact signal in immunomodulation [39,54]. The capacity of MSC to inhibit T cell proliferation was significantly reversed when Notch signalling was blocked through the addition of a Notch signalling inhibitor (N-(N-(3,5-difluorophenacetyl)-L-alanyl)-S-phenylglycine t-butyl ester (DAPT)) or a Jagged-1 neutralising antibody [40]. In support of these findings, the data described here show that suppression of Notch signalling by GSI resulted in a reduction of MSC driven expansion of Treg. As addition of GSI only partially abrogated the expansion of Treg in MSC co-cultures, we must consider the possibility that GSI is unable to inhibit all Notch signalling as previously discussed [55]. Furthermore, our study identified the specific Notch ligand responsible for this effect. Utilising Jagged-1 knock down MSC, we demonstrate that the expansion of Treg was significantly impaired. A recent study identified Notch-1 on CD4^+^ cells as the principle receptor involved in MSC recruitment of inducible Treg (iTreg)*.* This study utilised a GSI inhibitor and a neutralising Notch-1 antibody to demonstrate a role for Notch-1 signalling in MSC expansion of Treg in CD4^+^/MSC co-cultures [37]. However, as those authors started with a whole CD4^+^ T cell population (containing Foxp3^+^ CD4^+^ T cells), that study did not provide evidence for the capacity of MSC to induce a population of Treg from Foxp3^−^ T cells. Indeed, careful analysis suggests that these data [37] support our findings that MSC expand but do not induce a population of Treg and that Jagged-1 expression by MSC (Figure 1) and potential Notch-1 expression by CD4^+^ T cells [37] facilitates MSC expansion of Treg *in vitro*. Differences in cell populations and species (human versus mouse MSC) may also account for some of the disparities between these studies.

It is now known that MSC modulation of DC function involves both soluble and cell contact factors [20-23,56-58]. Our data clearly confirm that MSC are capable of inhibiting LPS-induced DC maturation and identifies an important role for Notch signalling in MSC expansion of Treg. The functionality of these semi-mature DC was examined further demonstrating an ability to inhibit antigen specific T cell proliferation in a Notch dependent manner, indicating regulatory properties. Interestingly, the semi-mature DC described herein were capable of Treg induction. Two previous studies support the importance of the Notch signalling pathway in immune regulation [22,23]*.* Using a GSI (DAPT), Li *et al.* demonstrated a key role for Notch signalling in MSC generation of regulatory DC [23]. Similar studies carried out using siRNA techniques showed that tDC regulation of lymphocyte proliferation was dependent on Jagged-2 signalling [22]. Following co-culture with MSC, the resulting tDC were found to express increased levels of Jagged-2; siRNA knock down of this ligand resulted in abrogation of tDC inhibition of lymphocyte proliferation [22]. Collectively, the data herein and that of others complete the story of Notch signalling in MSC modulation of T cells and DC*.* We showed that not only do Jagged-1 expressing MSC expand a Treg population, but in turn induce tDC which generate Treg from a null population.

Importantly, this is the first study to demonstrate the functional relevance for Jagged-1 signalling in MSC protection against inflammation *in vivo*. In agreement with previous findings, MSC reduced allergen-induced airway pathology and enhanced Treg [30,46,50] and IL-10 production in the lungs and spleen, respectively. The major advance in our understanding of MSC mechanisms of action *in vivo* lies in the observation that Jagged-1 knock down MSC failed to protect against allergic airway inflammation and was associated with reduced Treg in the lungs and decreased IL-10 production by allergen re-stimulated spleen cells. These observations have important ramifications for the Notch signalling family as potential therapeutic targets in MSC immunomodulation.

## Conclusions

In conclusion, our data demonstrated the requirement for Jagged-1 signalling in MSC expansion of Tregs and induction of semi mature DC *in vitro*. Importantly, these semi mature DC were functional and had the capacity to expand a population of Treg. For the first time, this study highlights the importance of Jagged-1 signalling in MSC protection *in vivo*. Utilising a mouse model of allergic airway inflammation, we show that MSC but not Jagged-1 knock down MSC provided protection against lung inflammation. Notably, the protective effects mediated by MSC were associated with enhanced Treg in the lung and significantly increased levels of IL-10 in splenocytes and these effects were not seen in mice treated with Jagged-1 knock down MSC. Together these findings enhance our understanding of the mechanisms of action used by MSC in promoting a regulatory environment through expansion/induction of semi mature DC and Treg. Moreover, linking *in vitro* data on MSC mechanism of action with *in vivo* models provides important pre-clinical data and informs us on how these cells may mediate their effects in relevant clinical diseases.

## Additional files

Additional file 1: Figure S1. Characterisation of MSC. BALB/c and C57BL/6 MSC were characterised for the expression of a variety of cell surface markers against their corresponding isotypes. In total, 10,000 events were recorded for each marker (A). MSC were co-cultured with splenocytes from BALB/c or C57BL/6 mice for 72 hours. Proliferation was measured by [^3^H] thymidine uptake over a six hour period. Mitogen driven proliferation was examined using ConA (5 ug/ml). Results are represented in counts per minute. MSC significantly reduced the proliferation of both allo-driven proliferation (**, *P* <0.01) and mitogen stimulation (***, *P* <0.001) (B). MSC (5 × 10^4^) were cultured for 21 days in control media or with osteogenic, or adipogenic medium. Cultures were stained with Alazarin Red and Oil Red O (C). MSC (2 × 10^5^) were also cultured in a control medium or chondrogenic medium for 21 days after which mRNA levels of the chondrogenic markers aggrecan and collagen II were examined by RT-PCR (D). Results are representative of multiple passages. Additional file 2: Figure S2. Notch/Jagged family expression profile and verification of Jagged 1 knockdown MSC. MSC were examined for expression of the Notch/Jagged family mRNA by RT-PCR. Known positive controls were used for each set of primers, Notch 1, Delta Like ligand 1 (spleen), Notch 2 (bone marrow), Jagged1 and Jagged 2 (J774) (A). Jagged 1 shMSC were positively selected using puromycin and allowed to reach confluence, the cells were passaged and 5 × 10^6^ cells were collected using trizol for mRNA analysis. RNA was isolated and cDNA produced. The cells were examined at a protein level or Jagged 1 expression by flow cytometry (B). The cells were also tested by real time PCR for the expression of Jagged 1 (C) and reverse transcription PCR for Delta like ligand 1 (D) and the results compared with non-transduced MSC, non-silencing control MSC or non-template control (NTC). Importantly, the Jagged 1 knock down MSC and the non silencing control MSC expressed Delta like ligand 1. Additional file 3: Figure S3. Characterisation of Jagged 1 knockdown MSC. BALB/c Jagged 1 knock down (J1 KD) MSC were characterised by looking at the expression of a variety of cell surface markers against their corresponding isotypes. In total, 10,000 events were recorded for each marker (A). J1 KD MSC (5 × 10^4^) were cultured for 21 days in control medium or with osteogenic, or adipogenic medium. Cultures were stained with Alazarin Red and Oil Red O (B). MSC (2 × 10^5^) were cultured in a control media or chondrogenic media for 21 days after which mRNA levels of the chondrogenic markers aggrecan and collagen II were examined by RT-PCR (C). Results are representative of similar findings at multiple passage. Additional file 4: Figure S4. MSC induce a semi mature DC phenotype. DC (0.5 × 10^6^/ml) matured with LPS (100 ng/ml) (mDC) displayed increased MHC class II and CD86 expression after 48 hours detected by flow cytometry. DC matured in the presence of MSC (1.5 × 10^5^/ml) had decreased levels of (A) MHC class II and (B) CD86 expression, indicative of a semi-mature phenotype. Bar charts represent mean fluoresecence intensity (MFI). Data represents three studies. (*, *P* <0.05) (***, *P* <0.001). C57BL/6 DC pulsed with I-Eα peptide were co-cultured with wild-type (Wt) allogeneic MSC or Jagged-1 knocked down (Jag 1^KD^) MSC for 48 hours. The number of DC presenting I-Eα peptide was measured using a YAe biotin conjugated anti-I-A^b^: Eα complex specific antibody. Percentages of cells are displayed within the marked regions (C).

## Abbreviations

DAPT: N-(N-(3,5-difluorophenacetyl)-L-alanyl)-S-phenylglycine t-butyl ester
DC: dendritic cells
Dlll1: delta like ligand 1
DMSO: dimethyl sulfoxide
ELISA: enzyme-linked immunosorbent assay
FBS: fetal bovine serum
GFP: green fluorescent protein
GSI: gamma secretase inhibitor
H & E: haematoxylin/eosin
iDC: immature dendritic cells
IFN: interferon
IL: interleukin
iTreg: inducible regulatory T cell
LPS: lipopolysaccharide
mDC: mature dendritic cells
MFI: mean fluorescence intensity
MHC: major histocompatibility complex
MSC: mesenchymal stromal cells
OVA: ovalbumin
PBS: phosphate-buffered saline
shRNA: short hairpin RNA
siRNA: small interfering RNA
tDC: tolerogenic dendritic cells
TGFβ: transforming growth factor beta
Treg: regulatory T cells
