# Supplementary figures and images for: Jagged-1 is required for the expansion of CD4+ CD25+ FoxP3+ regulatory T cells and tolerogenic dendritic cells by murine mesenchymal stromal cells
Source: Stem Cell Res Ther. 2015 Mar 11;6(1):19. doi: 10.1186/s13287-015-0021-5 (PMC4414370; doi:10.1186/s13287-015-0021-5)

## Slide 1
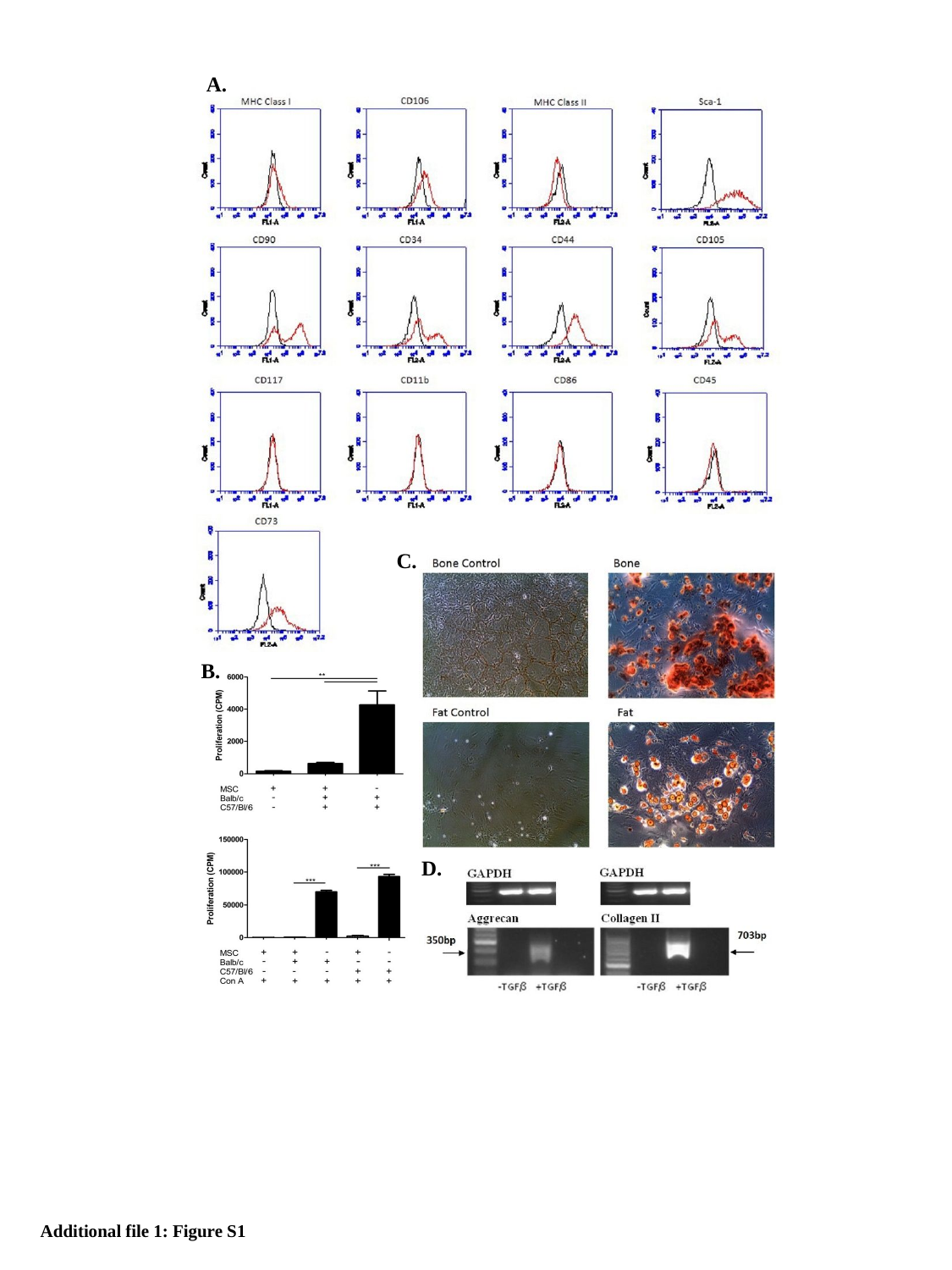

A.
C.
B.
D.
Additional file 1: Figure S1

Supplement: Additional file 1: Figure S1. — Characterisation of MSC. BALB/c and C57BL/6 MSC were characterised for the expression of a variety of cell surface markers against their corresponding isotypes. In total, 10,000 events were recorded for each marker (A). MSC were co-cultured with splenocytes from BALB/c or C57BL/6 mice for 72 hours. Proliferation was measured by [3H] thymidine uptake over a six hour period. Mitogen driven proliferation was examined using ConA (5 ug/ml). Results are represented in counts per minute. MSC significantly reduced the proliferation of both allo-driven proliferation (**, P <0.01) and mitogen stimulation (***, P <0.001) (B). MSC (5 × 10^4^) were cultured for 21 days in control media or with osteogenic, or adipogenic medium. Cultures were stained with Alazarin Red and Oil Red O (C). MSC (2 × 10^5^) were also cultured in a control medium or chondrogenic medium for 21 days after which mRNA levels of the chondrogenic markers aggrecan and collagen II were examined by RT-PCR (D). Results are representative of multiple passages. [file 13287_2015_21_MOESM1_ESM.pptx]

## Slide 1
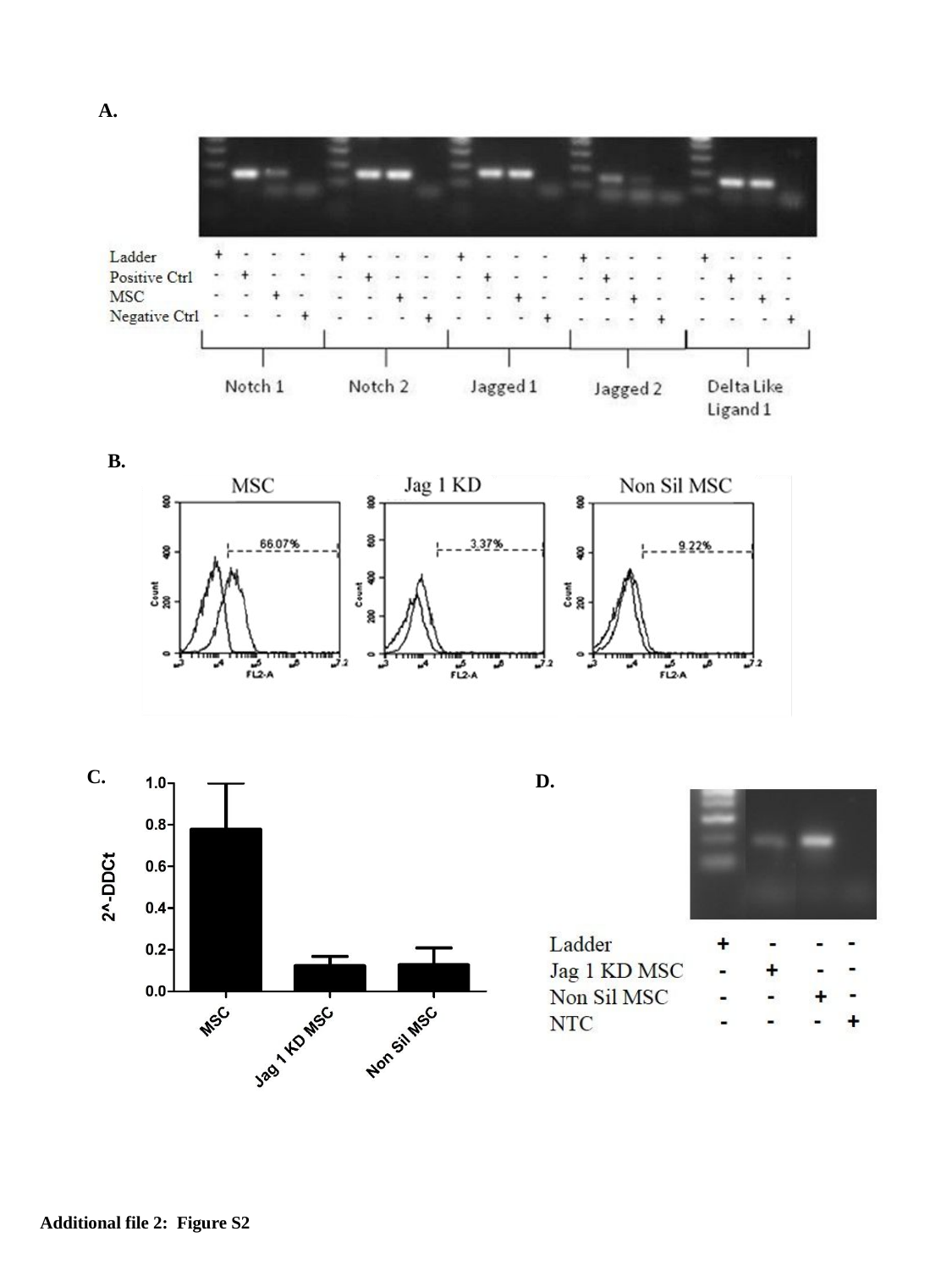

A.
B.
C.
D.
Additional file 2: Figure S2

Supplement: Additional file 2: Figure S2. — Notch/Jagged family expression profile and verification of Jagged 1 knockdown MSC. MSC were examined for expression of the Notch/Jagged family mRNA by RT-PCR. Known positive controls were used for each set of primers, Notch 1, Delta Like ligand 1 (spleen), Notch 2 (bone marrow), Jagged1 and Jagged 2 (J774) (A). Jagged 1 shMSC were positively selected using puromycin and allowed to reach confluence, the cells were passaged and 5 × 10^6^ cells were collected using trizol for mRNA analysis. RNA was isolated and cDNA produced. The cells were examined at a protein level or Jagged 1 expression by flow cytometry (B). The cells were also tested by real time PCR for the expression of Jagged 1 (C) and reverse transcription PCR for Delta like ligand 1 (D) and the results compared with non-transduced MSC, non-silencing control MSC or non-template control (NTC). Importantly, the Jagged 1 knock down MSC and the non silencing control MSC expressed Delta like ligand 1. [file 13287_2015_21_MOESM2_ESM.pptx]

## Slide 1
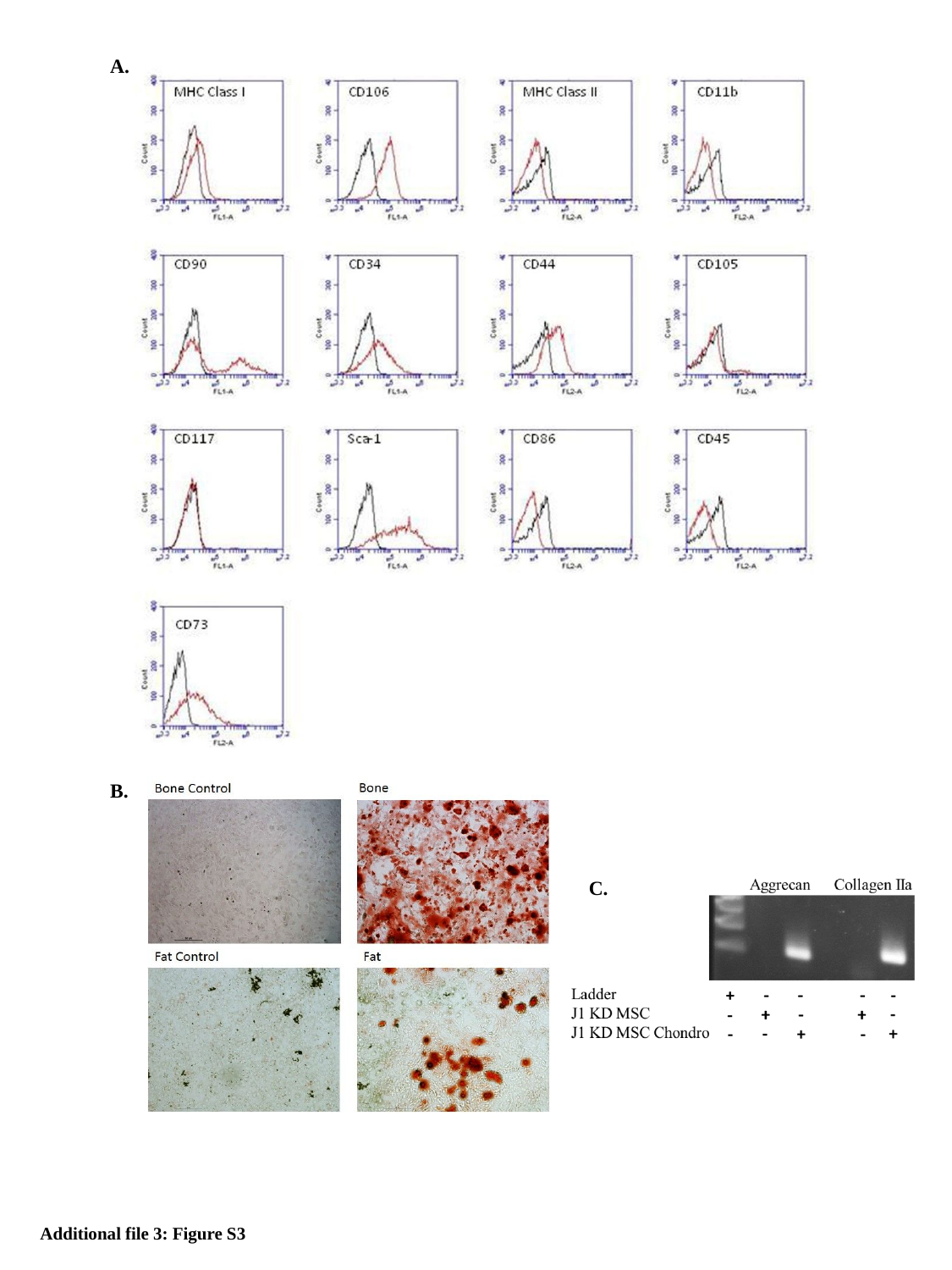

A.
B.
C.
Additional file 3: Figure S3

Supplement: Additional file 3: Figure S3. — Characterisation of Jagged 1 knockdown MSC. BALB/c Jagged 1 knock down (J1 KD) MSC were characterised by looking at the expression of a variety of cell surface markers against their corresponding isotypes. In total, 10,000 events were recorded for each marker (A). J1 KD MSC (5 × 10^4^) were cultured for 21 days in control medium or with osteogenic, or adipogenic medium. Cultures were stained with Alazarin Red and Oil Red O (B). MSC (2 × 10^5^) were cultured in a control media or chondrogenic media for 21 days after which mRNA levels of the chondrogenic markers aggrecan and collagen II were examined by RT-PCR (C). Results are representative of similar findings at multiple passage. [file 13287_2015_21_MOESM3_ESM.pptx]

## Slide 1
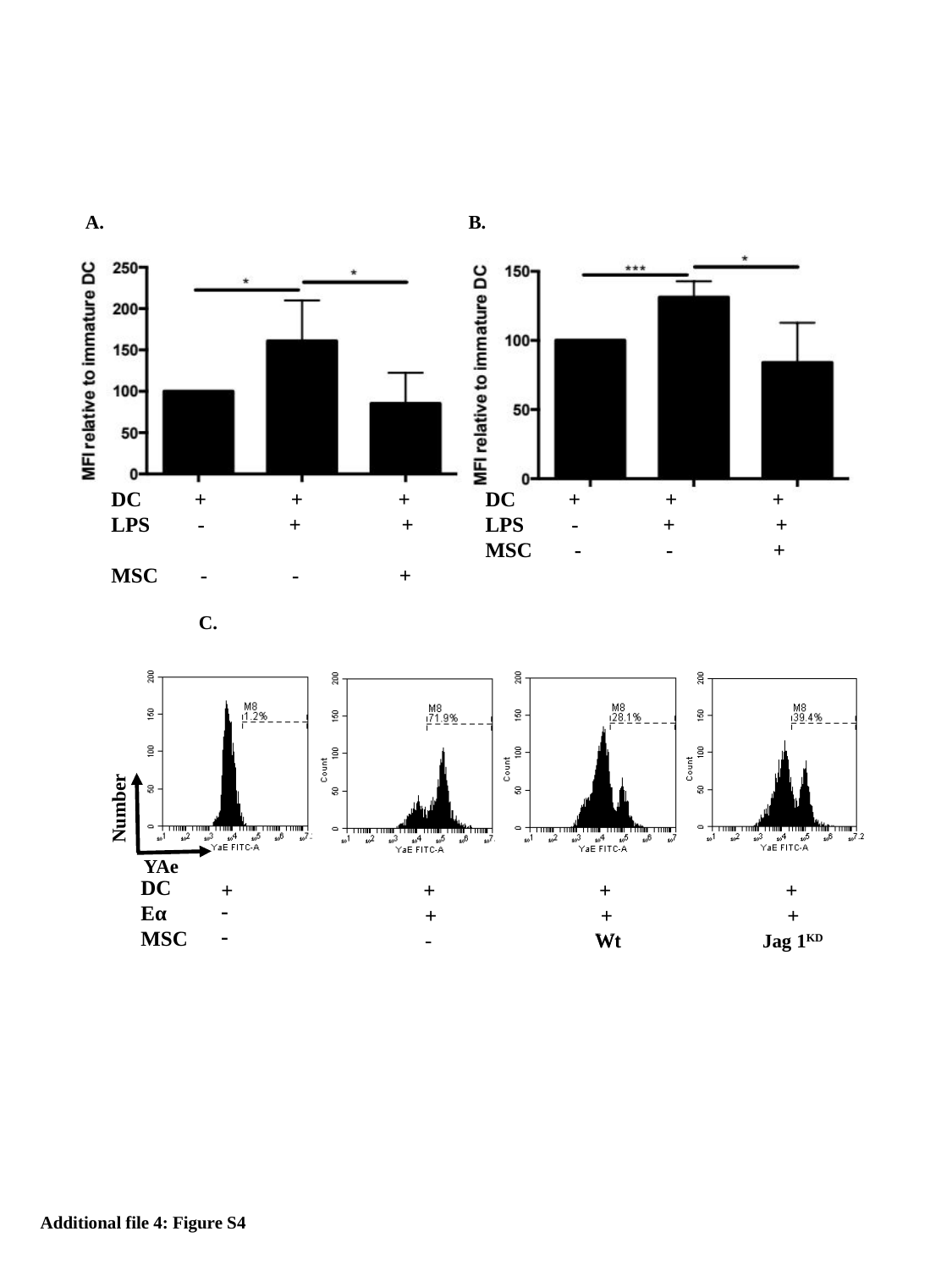

A.
B.
DC + + +
LPS - + +
MSC - - +
DC + + +
LPS - + +
MSC - - +
C.
Number
YAe
DC
Eα
MSC
Wt
Jag 1KD
+ + + +
 + + +
 - Wt
Additional file 4: Figure S4

Supplement: Additional file 4: Figure S4. — MSC induce a semi mature DC phenotype. DC (0.5 × 10^6^/ml) matured with LPS (100 ng/ml) (mDC) displayed increased MHC class II and CD86 expression after 48 hours detected by flow cytometry. DC matured in the presence of MSC (1.5 × 10^5^/ml) had decreased levels of (A) MHC class II and (B) CD86 expression, indicative of a semi-mature phenotype. Bar charts represent mean fluoresecence intensity (MFI). Data represents three studies. (*, P <0.05) (***, P <0.001). C57BL/6 DC pulsed with I-Eα peptide were co-cultured with wild-type (Wt) allogeneic MSC or Jagged-1 knocked down (Jag 1KD) MSC for 48 hours. The number of DC presenting I-Eα peptide was measured using a YAe biotin conjugated anti-I-Ab: Eα complex specific antibody. Percentages of cells are displayed within the marked regions (C). [file 13287_2015_21_MOESM4_ESM.pptx]
